# From jobs to careers: drivers and barriers to career development in emerging labor markets

**DOI:** 10.3389/fsoc.2024.1486871

**Published:** 2024-11-06

**Authors:** Wang Luwei, Ma Huimin

**Affiliations:** School of Accounting, Sichuan Technology and Business University, Meishan, China

**Keywords:** social network theory, career development, emerging labor markets, career barriers, social capital competing interests

## Abstract

**Introduction:**

This study aims to reveal the intrinsic and extrinsic drivers of career development in emerging labor markets and to explore the impact of these drivers and barriers on career development. With the rapid transformation of global industrial structure, career development in emerging industries such as artificial intelligence, big data, new energy and e-commerce is gradually attracting attention.

**Methods:**

This study utilizes a mixed research method of questionnaires and in-depth interviews. The research team distributed a total of 700 questionnaires to practitioners in China, the United States, Japan, Germany and India, and collected interview data from 20 industry practitioners. These data were analyzed from both quantitative and qualitative perspectives to analyze the drivers and barriers to career development, and structural equation modeling was used to analyze the relationship between career motivation, barriers, social networks, and career satisfaction.

**Results:**

The results indicate that career development motivation significantly and positively influences career satisfaction, while individuals with high career satisfaction perceive fewer career barriers. In addition, career barriers significantly influenced individuals’ perceptions of career discrimination. The study also found that social networks play an important supportive role in career development, and that extensive social networks increase career satisfaction. Individuals with high motivation were more resilient in the face of external barriers and were willing to retrain to improve their occupational skills.

**Discussion:**

To promote career development in emerging industries, labor market policies should optimize and create fair and inclusive work environments for emerging industries. By eliminating gender, age, and racial discrimination and providing employee support programs, career development satisfaction and opportunities can be effectively enhanced.

**Conclusion:**

Rapid technological updates, high work pressure, and cross-cultural barriers in emerging industries are the main challenges to career development today. This study suggests that governments and enterprises should jointly provide flexible vocational training and support policies to help practitioners adapt to the rapidly changing occupational environment.

## Graphical abstract

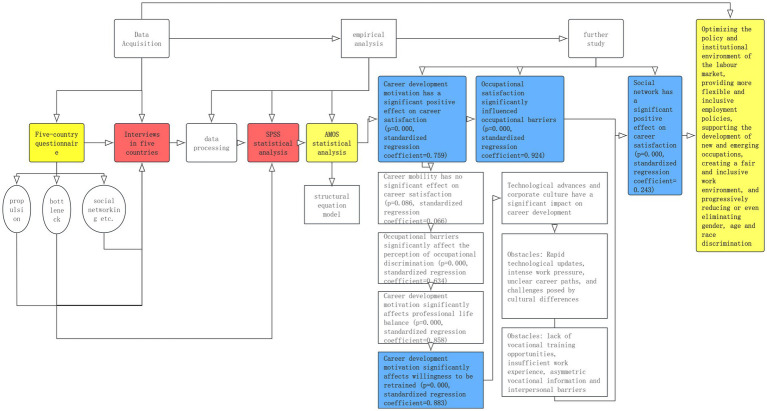

## Introduction

Career development encompasses a number of disciplinary areas such as psychology, sociology, economics, and education (Andes, 1986; Belch, 1991; Pannell, 1980). The match between personality and career environment is of importance for career success and well-being. From an interdisciplinary perspective, it is crucial to explore how these fields converge in shaping career trajectories. For example, psychological factors such as self-efficacy and motivation interact with sociological factors like social roles and institutional structures to influence career outcomes. Additionally, economic conditions and educational opportunities further modulate how individuals navigate their career paths. Career development can be categorized into five distinct phases of growing, exploring, establishing, maintaining, and exiting, and at each phase individuals need to address different career development tasks (Lee and Lee, 2021; Nagar, 2018), which play a key role in overall career satisfaction and success. These phases, while often linear, can overlap or repeat depending on the individual’s circumstances, highlighting the need for adaptive strategies in career management. For instance, during economic downturns, individuals might cycle back to the exploring phase as they seek new opportunities. This underscores the dynamic nature of career development tasks and their impact on long-term success. Individuals need to achieve a certain level of career maturity (Monteiro et al., 2019) during their career development in order to effectively respond to challenges in career choice and development, including decision making, realism of career choices, and readiness for career roles (Yeboah et al., 2020), such as navigating job rejections and adapting to new work environments. Career maturity involves not only the cognitive ability to make informed career choices but also emotional readiness to deal with uncertainties and setbacks. Individuals who possess higher career maturity are more likely to make realistic career decisions that align with their personal goals and market conditions, thus enhancing their satisfaction and long-term success. Furthermore, developing career maturity is a gradual process that requires individuals to continuously reflect on and adjust their career objectives. Individuals’ confidence in their ability to successfully complete a given task directly influences career choice and career behavior, for example, individuals with high confidence may pursue more challenging career paths. Additionally, outcome expectations, such as anticipated rewards, further influence their career motivation and effort (Molelekeng-Adeniran, 2022). This relationship between self-efficacy and career outcomes has been widely documented, suggesting that individuals who believe in their ability to succeed are more likely to pursue challenging career goals and persist in the face of difficulties. Moreover, the interaction between self-efficacy and outcome expectations creates a feedback loop where positive experiences reinforce confidence, leading to higher motivation and greater effort. Individuals give meaning and direction to the career development process by telling and reconstructing career stories, and there is an interactive relationship between individual subjective experience and career development (Ghosh et al., 2024; Laquiores et al., 2016). This narrative approach emphasizes the role of personal agency in career development. By reflecting on their experiences, individuals construct career stories that give coherence and meaning to their professional lives, helping them to make sense of past decisions and set future goals. This process of storytelling not only shapes individual career paths but also influences how others perceive and support their career aspirations. Individuals can enhance their human capital by investing in education and skills training, which leads to higher returns in the labor market, and education and training have a long-term impact on career development (Mutasa, 2019). In particular, investing in education and vocational training is essential in preparing individuals for the demands of an ever-changing labor market. As industries evolve and new technologies emerge, individuals must continuously update their skills to remain competitive. Education not only provides the technical skills necessary for specific job functions but also fosters critical thinking and problem-solving abilities that are essential for career progression. Individuals develop certain stable occupational values and motivations over the course of their careers, and these occupational anchors influence their career choices and career paths (Barbieri and Gioachin, 2022; Märkl et al., 2021). These occupational anchors, which include values such as autonomy, security, and creativity, provide a stable foundation for career decisions. Once established, they act as guiding principles that influence how individuals respond to career opportunities and challenges. Understanding one’s occupational anchors is crucial for long-term career satisfaction, as misalignment between personal values and career paths can lead to job dissatisfaction and burnout. Career development is complex and systemic in nature with the interaction of various environmental systems, such as microsystems (e.g., family and work environments), mesosystems (e.g., school and community), and macrosystems (e.g., culture and social policies) during an individual’s career development (Zhang et al., 2023; Zhang, 2024). This ecological approach underscores the fact that career development does not occur in isolation; rather, it is influenced by a variety of interconnected systems. For instance, family expectations and support may play a critical role in career decision-making, while broader societal factors such as economic policies and cultural norms also shape the opportunities available to individuals. Recognizing these multi-layered influences is essential for understanding the diverse experiences of career development across different social contexts.

Social network theory is mainly about how social structure affects behavior in individuals and groups. It looks at the pattern, structure, and dynamics of social relationships by showing how they affect things like the flow of information, the allocation of resources, the building of social capital, and the coordination of behavior (Hopewell et al., 2024; Ribner, 2020). Social networks act as conduits for career development, offering access to information, resources, and opportunities that might otherwise be unavailable. The strength and reach of an individual’s social network can significantly influence their career trajectory, as networks provide essential support and connections for job mobility and advancement. Analyzing the connections between nodes (individuals or organizations) in a network helps to understand how resources and information are transmitted and shared in the network, and individuals will have access to more novel and diverse information resources through looser chains of relationships (Koene and Pichault, 2021), facilitating innovation and development of individuals and organizations. In addition to providing resources, social networks can also shape individuals’ perceptions of career possibilities and influence career-related decision-making. For example, individuals who are part of high-density networks may experience pressure to conform to established career norms, while those in more diverse or loosely connected networks may have greater freedom to pursue unconventional career paths. Social networks provide structural conditions for individuals to acquire social capital, the quality and quantity of which will vary across different network structures, and in complex social systems, the behavior of individuals and groups often needs to be coordinated through network relationships (Duan, 2022). In particular, networks characterized by weak ties tend to provide more diverse information and opportunities, whereas strong ties facilitate trust and cooperation. Both types of connections are vital, as they offer complementary resources essential for long-term career success. In business management, internal social networks help cooperation and information sharing among employees, thus increasing organizational efficiency and innovation. Analyzing the role of key nodes and bridges in the network can reveal which individuals or small groups play a central role in behavioral coordination (Fasone and Pedrini, 2023). Such networks not only enhance communication within organizations but also enable the exchange of tacit knowledge, which is often critical for innovation and problem-solving. Identifying key nodes within these networks—those individuals who act as hubs for information flow—allows organizations to better manage resources and streamline coordination efforts. With the rise of social media, the way individuals interact with each other and the path of information dissemination have changed profoundly, and attention needs to be paid to the diffusion of information, opinion formation, and social influence in the new types of networks (Rahbari et al., 2023). The proliferation of online platforms has expanded the reach of social networks, creating new dynamics in how career information is shared and how individuals position themselves within their professional circles. In this new digital context, social capital has become more fluid, and individuals must navigate these platforms strategically to enhance their career prospects.

Emerging labor markets represent a structural transformation, mainly in the form of a shift in the center of gravity of the economy from the traditional industrial sector to services and high-tech industries (Glebova et al., 2024; Sattich et al., 2024; Seibert et al., 2024). This shift has significant implications for workforce skills and labor market dynamics. As traditional industries decline, there is a growing emphasis on specialized skills related to information technology, biotechnology, and artificial intelligence. These emerging industries require workers who not only possess technical expertise but also the ability to continuously adapt to technological advancements. The demand for labor in emerging fields such as information technology, artificial intelligence, and biotechnology has increased dramatically, while the gradual decline in the share of manufacturing and traditional services has set higher standards for skill requirements, leading to a rapid rise in the demand for a highly skilled and educated workforce, exacerbating income inequality and social divisions (Ferreira, 2024). This dual challenge of rising skill demands and widening social inequality presents new hurdles for career development, particularly for individuals in lower-income brackets who may lack access to the necessary educational resources or training opportunities. Globalization and digitization have sped up, leading to a more flexible labor market with trends like the gig economy. Freelancing, and remote work. It provides more employment opportunities for the workforce. But also brings with it income instability and a lack of social security, which makes these workers more vulnerable in the face of economic fluctuations and can also lead to increased burnout and mental health problems(Chwastek and Mynarska, 2024; Xu et al., 2023). Although flexible work arrangements have created more job opportunities, particularly for younger and highly mobile workers, they have also increased economic uncertainty for many. Without the traditional security of stable employment, individuals must contend with fluctuating incomes and the absence of benefits like health insurance and pensions, which can exacerbate mental health challenges. On the one hand, the global layout of multinational corporations has brought about international labor mobility, making some countries and regions destinations for outsourcing and production transfer, and exacerbating the imbalance in the global labor market. On the other hand, emerging economies such as China, India, and Southeast Asian countries have attracted large amounts of international investment and business expansion due to their large labor resources and relatively low labor costs (Been and Keune, 2022). These shifts in global labor distribution underscore the need for more adaptive and flexible educational and vocational training systems that can equip individuals with the skills necessary for success in a globalized, high-tech economy. Moreover, regional disparities in labor market development may deepen as lower-income countries struggle to upgrade their workforce capabilities to meet the demands of emerging industries. These changes will result in increased geographic economic disparities and problems of overutilization of the labor force in low-income countries, increasing demand for emerging skills such as data analytics and programming in high-technology industries, and pressure on traditionally skilled laborers to upgrade their skills, placing greater demands on the education and training system (Morris, 2021). As can be seen, the characteristics of emerging labor markets cover a wide range of aspects such as structural transformation, flexibility and uncertainty, globalization and geographical imbalances, and technology-driven and skills gaps (de Frutos Madrazo et al., 2012; Fasone and Pedrini, 2023; Jung, 2021; Kim, 2016; Ruane, 2013; Scovronick et al., 2010; Song, 2009), which is of great guiding significance for the formulation of rational labor policies, optimizing the allocation of human resources, and promoting sustainable economic development. Effective policy interventions will need to focus on addressing the widening skills gap, ensuring equitable access to educational opportunities, and creating systems that support continuous learning and skill upgrading throughout an individual’s career.

Motivation, as an internal force that drives individual actions and decisions, is usually closely related to individual needs, expectations and goals, and is divided into two categories: intrinsic motivation and extrinsic motivation (Zhang, 2024). Intrinsic motivation stems from an individual’s interest and satisfaction in the activity itself, while extrinsic motivation stems from external rewards or social evaluations (Seibert et al., 2024). While both types of motivation are important, intrinsic motivation has been shown to play a critical role in sustaining long-term engagement and innovation in one’s career. Individuals driven by personal interest and passion are more likely to persist in the face of obstacles and continue to invest in their professional growth. Intrinsic motivation promotes an individual’s sustained engagement and innovativeness in a task, whereas extrinsic motivation motivates behavior in the short term but may negatively affect long-term performance (Sattich et al., 2024; Zhang et al., 2023). However, relying solely on extrinsic rewards can diminish intrinsic interest, leading to lower overall job satisfaction over time. Organizations must strike a balance between providing external incentives and fostering environments that support personal growth and fulfillment. Factors such as leader support, teamwork, and career development opportunities significantly affect employees’ motivation levels, and employees show higher job motivation and job satisfaction when they perceive leader support and recognition, reflecting the moderating effect of the organizational environment on individual motivation (Medici, 2024). Leadership plays a crucial role in cultivating a motivating work environment. Leaders who recognize and support employees’ career aspirations contribute not only to higher motivation but also to increased loyalty and organizational commitment. Positive feedback and recognition from leaders are important factors that enhance employees’ intrinsic motivation, boosting their motivation and also their organizational commitment and job performance. In addition, opportunities for professional development and career advancement are essential for sustaining long-term employee engagement, as individuals are more likely to invest in their roles when they see a clear path for growth. Key factors of job design such as task challenge, job autonomy, and opportunities to use skills have been shown to have a significant effect on employee motivation, and a challenging and autonomous job design enhances employees’ intrinsic motivation and increases their job satisfaction and performance (Zhang et al., 2023).

Opposite to motivation are barriers, which act as impediments to goal attainment and tend to weaken motivation and lead to setbacks and failures for individuals or organizations. Barriers can be intrinsic, such as an individual’s low self-efficacy and lack of psychological resources (Glebova et al., 2024; Young and 이미나, 2022); or extrinsic, such as resource constraints and environmental uncertainty (Wu, 2021). Low self-efficacy can lead to avoidance of difficulties and weakening of coping strategies, which can further diminish motivation. Self-efficacy is a central factor influencing individuals’ actions and decision-making, and when individuals feel that they are unable to effectively cope with challenges, they experience decreased motivation and task avoidance, which in turn affects their job performance and personal development (Morris, 2021; Zaman et al., 2020). In addition, external barriers such as insufficient resources and environmental changes can have a direct impact on employee productivity and satisfaction (Kumar et al., 2021). Organizations, when faced with insufficient resources, must respond to the challenges of the external environment by adapting their internal structures and strategies, and the enhancement of motivation and overcoming of obstacles are not only dependent on the individual’s intrinsic traits, but are also influenced by the organizational environment and external conditions. Thus, a well-designed organizational environment can mitigate some of the adverse effects of external challenges by fostering resilience and adaptability among employees. Individuals undergo complex psychological and behavioral processes when confronted with intrinsic motivation and external barriers, and with high intrinsic motivation, individuals are more likely to take positive actions and coping strategies and show higher resilience and adaptability when confronted with external barriers (Ghosh et al., 2024; Ribner, 2020). Conversely, a lack of extrinsic motivation and an increase in barriers may lead to reduced motivation in individuals, which may affect their job performance and goal attainment (Fowee Gasaway and Alexeenko, 2024). To effectively manage these challenges, organizations should implement comprehensive support systems that address both individual and systemic barriers, ensuring that employees remain engaged and motivated despite external pressures.

Understanding the motivations and barriers to career development in the emerging labor market is important to help individuals adapt to the rapidly changing occupational environment, to promote the efficient allocation of human resources, and to develop sound labor policies. In particular, the understanding of how internal and external dynamics affect career development and how barriers impede career development are important to focus on. Studying career development in emerging labor markets by combining the perspective of social network theory provides a more comprehensive understanding of the role of internal and external dynamics in emerging labor markets, which can help to improve career development effectiveness and career satisfaction. The analysis of barriers can provide a reference for the development of effective career development strategies to help individuals and organizations overcome difficulties and achieve career goals. Therefore, future research should emphasize the importance of integrating personal, organizational, and societal factors when analyzing career development in rapidly evolving labor markets. Such an integrated approach will enable more effective interventions that address the complex challenges faced by both individuals and organizations in navigating the new realities of the labor market.

## Materials and methods

### Materials

A total of 700 questionnaires were distributed to China (350) and abroad (100 in the United States, 75 in Japan, 75 in Germany, and 100 in India), with a focus on emerging industries, including artificial intelligence and big data (175), new energy and environmental protection (175), e-commerce and sharing economy (175), and modern agriculture and healthcare (175), in order to reflect these industries’ characteristics of career development. The distribution of questionnaires was designed to ensure a representative sample across these five countries, considering their different levels of economic development and the characteristics of their respective emerging labor markets. These countries were selected based on their significant role in global economic trends, as well as their unique labor market dynamics that make them ideal for studying career development in emerging industries. The sample size in each country was determined by population size, industry relevance, and research feasibility. Furthermore, the selected industries were chosen because they represent sectors undergoing rapid growth and transformation, offering valuable insights into the career development processes in dynamic labor markets. Interviews were conducted with a total of 20 people, 10 in China and 10 abroad, choosing five countries, namely, China, the United States, Japan, Germany and India, covering different levels of economic development and emerging labor market characteristics, which helps to analyze the characteristics of the emerging labor market and its motivation and obstacles to career development in different cultural contexts. The interviewees were selected through a purposive sampling method, ensuring that participants held relevant positions in key industries and represented different stages of career development. This allowed for a balanced perspective on the motivational factors and barriers encountered in both established and emerging economies. The use of interviews complemented the quantitative data by providing deeper insights into individual experiences and contextual factors that influence career development. To ensure the validity and reliability of the research, the questionnaire design followed a rigorous pilot testing phase. Prior to full-scale distribution, the questionnaire was tested with a small sample of industry professionals to assess clarity, relevance, and overall structure. Feedback from the pilot test was used to make necessary adjustments, ensuring that the final questionnaire was both comprehensive and easy to understand for respondents across different cultural and linguistic backgrounds. This step was crucial in minimizing response bias and improving the accuracy of the data collected. 689 questionnaires were collected and 683 valid questionnaires were obtained by eliminating the non-compliant questionnaires, and the questionnaire recovery rate and validity rate reached the standard. Non-compliant questionnaires were excluded based on predefined criteria, including incomplete responses, inconsistencies in the answers, or failure to meet the demographic or industry-related inclusion criteria. This ensured that only high-quality data were included in the final analysis. The high response rate and validation process reflect the robustness of the data collection method.

### Methods

This study employs a mixed research methodology that combines quantitative and qualitative approaches in order to provide insight into the drivers and barriers to career development in an emerging labor market. The quantitative component, represented by the questionnaire, was designed to capture a broad spectrum of variables influencing career development, while the qualitative interviews were used to explore these variables in greater depth, providing context and meaning to the statistical findings. The core of the study is the collection of data through questionnaires and in-depth interviews to analyze the research questions from multiple perspectives. In terms of sample selection for the quantitative research, a stratified random sampling method was employed to ensure that different subgroups within the population were adequately represented. The population was divided into strata based on key demographic variables such as age, gender, and education level, as well as industry and job role, to capture a diverse range of experiences and perspectives on career development. This approach not only enhanced the representativeness of the sample but also allowed for more detailed subgroup analyses in the results, offering insights into how different demographic and occupational groups navigate career development in emerging labor markets. The questionnaire portion of the study utilized a structured questionnaire that was designed to take into account a variety of career development related factors such as motivation for career development, career barriers, social networks, and career satisfaction (da Costa Oliveira Caldas, 2020; Fowee Gasaway and Alexeenko, 2024; Kyoung-phil, 2017; Laquiores et al., 2016). The questions were carefully crafted to ensure clarity and relevance across different cultural contexts, using language that was accessible and culturally neutral to avoid misinterpretation. Furthermore, the questionnaire included both closed-ended questions to facilitate quantitative analysis and a few open-ended questions to capture additional qualitative insights from respondents. The questionnaire data were statistically analyzed using SPSS and AMOS software to construct a structural equation model of motivation and barriers to career development, and to test the path relationships between the variables and their significance. This statistical analysis enabled the researchers to identify key predictors of career satisfaction and barriers, as well as the mediating and moderating effects of various factors such as social networks and motivation. The structural equation modeling approach was particularly well-suited to this study, given the complexity of the relationships between the variables under investigation. The themes related to career development motivation and barriers were identified and extracted by repeatedly reading the interview texts, categorizing similar themes, and comparing the views of different interviewees to find commonalities and differences.

This study strictly adhered to the ethical norms of academic research. During the questionnaires and interviews, all participants were informed of the purpose and content of the study and their informed consent was obtained. In addition, participants were assured of their anonymity and the confidentiality of their responses. The study complied with all applicable regulations regarding data protection and privacy, and all data were anonymized prior to analysis to protect the identities of the participants. All data were anonymized during the study to ensure participants’ privacy and data security.

## Results

### A review of social network theory

**Figure 1:**
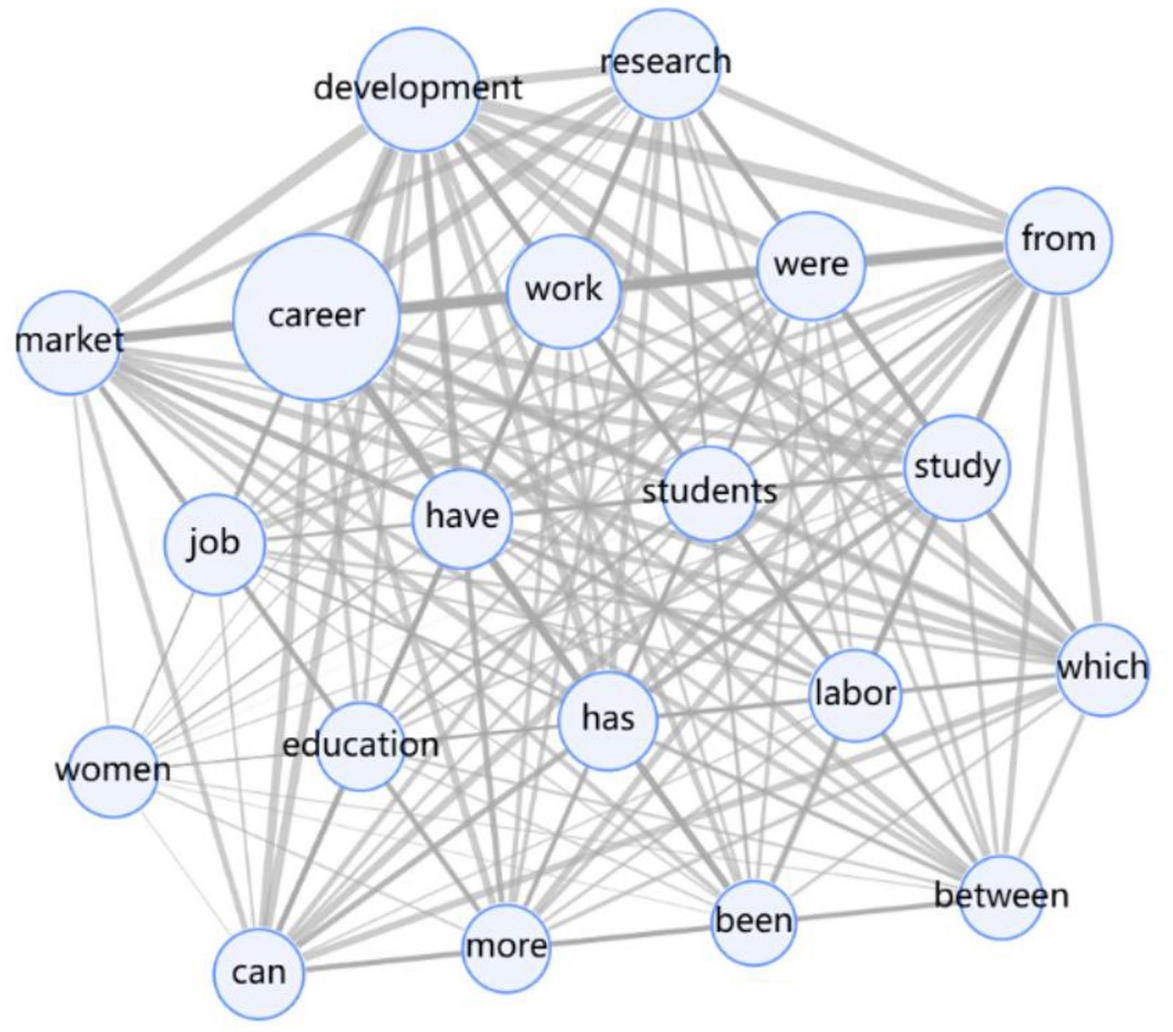
Literature social network relationships.

Network size is a measure of the number of nodes in a social network, and the size of the network directly affects an individual’s ability to access information and resources (Rahbari et al., 2023). In the process of career development, individuals with larger social networks are more likely to obtain diverse information and resources, which is conducive to increasing opportunities for career development. As shown in Figure 1, the “career” node is at the center of the network and is connected to multiple nodes. Individuals at the core of the career development process usually have a wide range of social networks, through which they can obtain important information about job vacancies, vocational training, and industry trends.

Network density refers to the ratio of the number of relationships that actually exist in a network to the maximum number of relationships that could exist, with higher network density indicating stronger connections between individuals (Dunbar, 2024; Won, 2022). High-density networks contribute to the rapid dissemination of information and sharing of resources, but may also lead to homogenization of information, which is not conducive to innovation and diversification. The connectivity between the nodes in Figure 1 is very dense, with closer connections between individuals and high information mobility during the career development process. It is important to note that this dense network may also limit individuals’ access to novel and diverse information, affecting the innovativeness of career development. Centrality is a measure of an individual’s importance in a network and is divided into degree centrality, proximity centrality, and mediated centrality. Degree centrality reflects the number of direct connections an individual has with other individuals (Hirst, 2023; Kilelu et al., 2022); proximity centrality reflects an individual’s average distance from all other individuals; and mediated centrality reflects an individual’s average distance from all other individuals in the network.; mediating centrality reflects how often an individual acts as a bridge between other individuals. The “career” node in Figure 1 has high proximity centrality, which is at the core of the career development network and maintains direct contact with multiple nodes, which is conducive to the acquisition of resources and the dissemination of information. Nodes with high proximity centrality can quickly contact other nodes in the network, shorten the information transmission path and improve the information flow efficiency. Nodes with high intermediary centrality play the role of bridge between other nodes and promote the exchange of information and resources between different groups. Key nodes are individuals with high intermediary centrality in the network, and these individuals play a central role in career development. Due to their high centrality, “career” nodes are able to influence the information flow and resource allocation of the whole network. Key nodes can not only directly influence career development by providing career opportunities and resources, but also indirectly promote career development by connecting different social groups and facilitating the sharing of information and resources. Bridging roles are individuals who connect different sub-networks and are able to integrate and transfer information and resources from different groups (An, 2024; Koene and Pichault, 2021). In the process of career development, bridging roles help individuals break through the bottleneck of career development and realize career transition through their unique position. The nodes of “development,” “education,” and “labor” in Figure 1 are such bridging roles, which connect different. They connect different career development resources and opportunities, and promote diversity and flexibility in career development. Individuals often use social networks to access resources such as job information, career advice, and skills training during the career development process. In the job search process, letters of recommendation and internal referrals are often more effective than open recruitment, reflecting the important role of social networks in career development. Social networks can also provide emotional support and psychological resources to help individuals cope with the pressure and challenges of career development. In the face of career bottlenecks and dilemmas, support from social networks can enhance the confidence and motivation of individuals, and promote the continuous advancement of their career development.

### Structural equation modeling analysis

**Figure 2:**
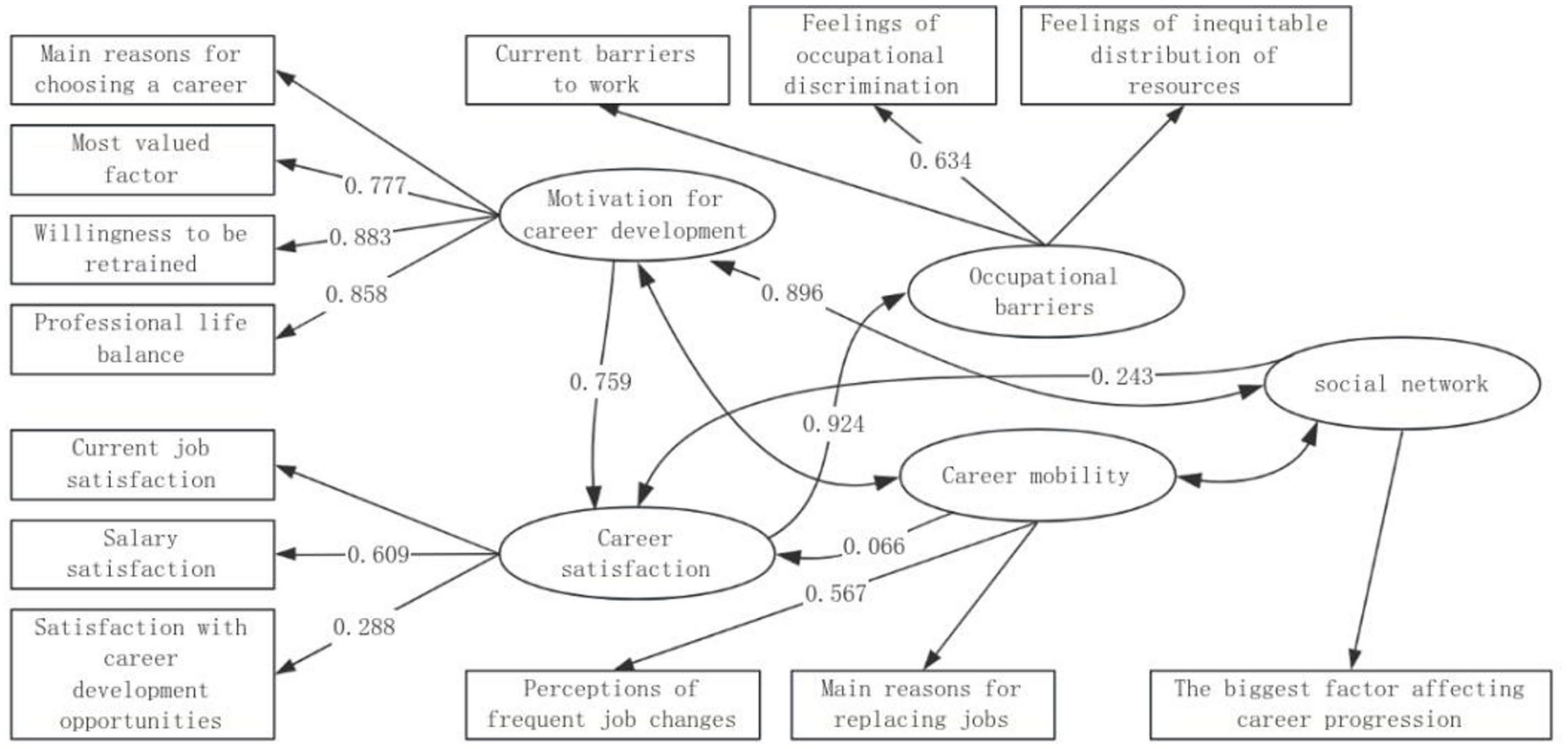
Structural equation model. 0.066 is significant at the 10% level, 0.567 is significant at the 5% level, and the rest labeled are significant at the 1% level. Those not labeled are not significant or not applicable.

Table 1 shows that career development motivation has a significant positive effect on career satisfaction (*p* = 0.000, standardized regression coefficient = 0.759), and the stronger an individual’s career development motivation, the higher his or her career satisfaction. This finding supports the importance of career development motivation in career planning and suggests that organizations should focus on stimulating employees’ career development motivation to enhance their career satisfaction when formulating human resource policies. Career satisfaction significantly affects career barriers (*p* = 0.000, standardized regression coefficient = 0.924), and individuals with high career satisfaction perceive fewer career barriers. Career satisfaction also significantly influenced career development opportunity satisfaction (*p* = 0.000, standardized regression coefficient = 0.288) and salary satisfaction (*p* = 0.000, standardized regression coefficient = 0.609), further confirming the centrality of career satisfaction in the career development process. Enhancing career satisfaction not only helps to reduce career barriers, but also enhances individual satisfaction with career development opportunities and pay, thus promoting career development. Social network has a significant positive effect on career satisfaction (*p* = 0.000, standardized regression coefficient = 0.243), and a broad and effective social network can enhance individuals’ career satisfaction. Social network not only provides career information and resources, but also provides an important support system for career development. For individuals, establishing and maintaining a good social network is an important strategy for career development; for organizations, encouraging employees to participate in industry exchanges and occupational social activities helps to enhance their social network, which in turn enhances career satisfaction. Occupational mobility does not have a significant effect on career satisfaction (*p* = 0.086, standardized regression coefficient = 0.066), but it has a significant positive effect on perceptions of frequent job changes (*p* = 0.033, standardized regression coefficient = 0.567), and individuals with high occupational mobility have a more positive attitude toward frequent job changes. Occupational mobility also significantly influenced the main reason for changing jobs (standardized regression coefficient = 0.764), and understanding the motivations and influences on occupational mobility in a labor market with frequent occupational changes is essential to help individuals make more informed career decisions. Occupational barriers significantly affect the perception of occupational discrimination (*p* = 0.000, standardized regression coefficient = 0.634), and individuals who perceive occupational barriers are more likely to perceive occupational discrimination, suggesting that organizations should pay attention to and reduce barriers encountered by their employees in their career development, and in particular, avoid and eliminate occupational discrimination. Occupational barriers also have a significant effect on current job barriers (standardized regression coefficient = 0.719), individuals who feel occupational barriers strongly also encounter more barriers in their current jobs, and organizations should take active measures to reduce the barriers of employees’ career development in order to enhance their job satisfaction and career satisfaction. Career development motivation also significantly affects individuals’ career life balance (*p* = 0.000, standardized regression coefficient = 0.858) and willingness to receive retraining (*p* = 0.000, standardized regression coefficient = 0.883), and individuals with strong motivation for career development are better able to find a balance between their career and their life and are willing to improve their career skills through retraining. Career-life balance is an important component of career satisfaction and career success, while willingness to retrain is key to adapting to the rapidly changing labor market. Therefore, motivating employees to develop their careers is essential to promote their career development and professional life balance. The results in Table 2 show that the model passed the tests for chi-square degrees of freedom ratio, GFI, RMSEA, RMR, CFI, NFI, and NNFI. Figure 2 shows the tested structural equation model.

**Table 1:** Summary of model regression coefficients.

X	→	Y	Unstandardized regression coefficients	SE	z (CR value)	*p*	Standardized regression coefficient
Motivation for career development	→	Career satisfaction	1.362	0.057	23.697	0.000	0.759
Career satisfaction	→	Occupational barriers	0.681	0.036	19.144	0.000	0.924
Career mobility	→	Career satisfaction	0.149	0.086	1.718	0.086	0.066
social network	→	Career satisfaction	0.404	0.051	7.915	0.000	0.243
Motivation for career development	→	Professional life balance	1.942	0.138	14.044	0.000	0.858
Motivation for career development	→	Willingness to be retrained	2.067	0.145	14.206	0.000	0.883
Motivation for career development	→	Most valued factor	1.619	0.120	13.447	0.000	0.777
Motivation for career development	→	Main reasons for choosing a career	1.000	–	–	–	0.513
Career satisfaction	→	Satisfaction with career development opportunities	0.297	0.040	7.385	0.000	0.288
Career satisfaction	→	Salary satisfaction	0.577	0.034	17.122	0.000	0.609
Career satisfaction	→	Current job satisfaction	1.000	–	–	–	0.847
Occupational barriers	→	Feelings of inequitable distribution of resources	0.070	0.048	1.483	0.138	0.062
Occupational barriers	→	Feelings of occupational discrimination	0.718	0.048	14.799	0.000	0.634
Occupational barriers	→	Current barriers to work	1.000	–	–	–	0.719
Career mobility	→	Perceptions of frequent job changes	0.847	0.396	2.137	0.033	0.567
Career mobility	→	Main reasons for replacing jobs	1.000	–	–	–	0.764
social network	→	The biggest factor affecting career progression	1.000	–	–	–	0.606

→ indicates a regression effect relationship or a measurement relationship; the bar “–” indicates that the item is a reference item.

**Table 2:** Indicators of model fit.

Common indicators	χ^2^	*df*	*p*	Chi-square (math.) degree-of-freedom ratio χ^2^/*df*	GFI	RMSEA	RMR	CFI	NFI	NNFI
Judgment criteria	–	–	>0.05	<3	>0.9	<0.10	<0.05	>0.9	>0.9	>0.9
Value	104.150	58	0.000	1.796	0.977	0.034	0.027	0.986	0.969	0.981
Other Indicators	TLI	AGFI	IFI	PGFI	PNFI	PCFI	SRMR	RMSEA 90% CI		
Judgment criteria	>0.9	>0.9	>0.9	>0.5	>0.5	>0.5	<0.1	–		
Values	0.981	0.964	0.986	0.623	0.720	0.733	0.032	0.023 ~ 0.045		

Default Model when *χ*^2^(78) = 3353.428, *p* = 1.000.

### Interview probes

A variety of motivations drive career development in the emerging labor market, and interviews show that both in China and abroad, individuals show strong intrinsic drive and extrinsic motivation in their career development. Individual career goals and pursuits are one of the most important motivators for career development, and rapid technological advances also provide a strong impetus for career development. Mr. Zhang, a senior data analyst from Alibaba, said he chose to enter the AI and big data industry because he is passionate about data mining and machine learning, and because he wants to change the world through the power of technology. Similarly, Sara Johnson, a senior software engineer from Google, mentioned that her career goal is to become an exceptional technologist and drive the company’s cutting-edge research in AI. Ms. Li, a mid-level engineer at BYD, a new energy company in China, noted that new energy technologies are evolving at a very fast pace, with new technologies and products emerging all the time, which forces her to keep learning and updating her knowledge in order to stay competitive. Max Müller, a R&D engineer at Bosch Germany, similarly emphasized that technological advances have made work more interesting and challenging, which has not only enhanced his job satisfaction but also inspired him to pursue career development. Corporate culture and development strategy play a key role in career development. Manager Wang, a human resource manager at Jingdong, mentioned that the company focuses on employees’ career development and internal promotion mechanism, and helps them realize their personal goals through regular training and career development planning. Rajesh Kumar, an industry expert at Tata Group in India, said that the company’s innovative culture and global development strategy provide employees with a broad career development platform, enabling them to accumulate experience in different countries and regions and expand their career horizons. Emily Brown, a junior employee at Amazon, mentions that through the company’s global business, she has the opportunity to participate in multinational projects and work with colleagues from different countries, which has enhanced her professional skills and expanded her international perspective. Hiroshi Tanaka, a mid-level manager at SoftBank Group in Japan, noted that the company’s international business has given him the opportunity to work overseas and gain international experience, which has given him a new impetus for his career development.

Mr. Chen, a junior employee at Huawei in China, said that the technology industry is very fast-changing and often requires constant learning and adapting to new technologies, which puts a lot of pressure and challenges on him. Michael Lee, a mid-level engineer at Microsoft Corporation in the United States, also mentioned that he felt the need to constantly update his skills to adapt to the rapid changes in technology, or else he risked becoming obsolete. The pressure brought by technological updates affects employees’ career development and increases anxiety and uncertainty at work. Ms. Li, an ordinary employee at Alibaba, mentioned that the company’s intense work pace and competitive pressure made her feel exhausted and even affected her physical and mental health. Hans Schmidt, a senior manager at BMW Germany, said that the company’s development prospects are good, but the pressure faced by top managers is also very high. According to him, they often need to work overtime and deal with complex problems, which to some extent limits his dedication and attention to career development. Ms. Wang, a mid-level employee at Jingdong, said that although the company provides many training opportunities, she is still confused about the direction of her career development and does not know how to achieve career advancement within the company. Anjali Singh, an industry expert at Infosys India, also mentioned that although the company has many development opportunities, the lack of clear career development planning and guidance makes employees feel overwhelmed in the process of career development. John Smith, a junior employee at Amazon, mentioned that when he was involved in multinational projects, he often encountered difficulties in communication and cooperation due to cultural differences, which not only affected the progress of the projects, but also limited his career development. Yuki Nakamura, a mid-level manager at Sony Corporation in Japan, also pointed out that the work culture and management styles in different countries are very different, which makes her face many adaptation and communication challenges when working overseas. It can be seen that the barriers to career development in emerging labor markets mainly include rapid technological updates, high work pressure, unclear career paths, and challenges posed by cultural differences.

## Discussion

Employees’ expectations on career development are diversified and complementary, and the proportion of choosing position promotion, skill enhancement, job stability, income increase, realizing personal value and others are 71.74, 55.05, 66.18, 85.80, 48.17, and 9.66%, respectively. They not only hope to get position promotion and skill enhancement in the workplace, but also attach importance to job stability and income increase, and the need to realize personal value cannot be ignored. At different stages and under different circumstances, employees may focus on these expectations differently. For example, in an economic downturn, job stability may be more important than job advancement, while in the early stages of an individual’s career, skills upgrading and job advancement may be the main goals.

Regarding the barrier of insufficient vocational training opportunities, a total of 316 people chose this option, accounting for 46.27% of the total. It can be seen that close to half of the respondents consider the shortage of training opportunities as one of the main barriers to their career development. Limited access to training may lead to employees’ skills not being updated sufficiently to keep up with industry developments, affecting both career advancement and personal growth. Insufficient investment in training by enterprises may be the main reason for this problem, while insufficient personal initiative and difficulties in accessing training resources may also be important factors contributing to this phenomenon. Insufficient work experience was mentioned by 258 people as a barrier to career development, accounting for 37.77%. Insufficient work experience tends to limit an individual’s development in the workplace because many positions require experience, and young employees or recent graduates are particularly vulnerable to this barrier. They may face more challenges when looking for jobs due to their lack of practical work experience, which affects their employment opportunities and limits their room for career development. Career information asymmetry affects 468 or 68.52% of the total number of students. Career information asymmetry is the problem of unequal information between job seekers and employers. This asymmetry may result in job seekers not being able to obtain true and accurate job information and make unfavorable career choices, and employers may miss out on good talents because they cannot accurately obtain the real situation of job seekers. The development of the Internet and social media has alleviated this problem to some extent, but asymmetry still exists due to the complexity and diversity of information. The most notable is interpersonal relationship disorder, with 628 people choosing this option, accounting for 91.95%. Good interpersonal relationship not only helps individuals integrate in the workplace, but also provides more opportunities for career development. Interpersonal relationship barriers may stem from personality traits, insufficient interpersonal communication skills, or the influence of the work environment. The prevalence of this issue shows that the development of soft skills and the establishment of interpersonal networks are particularly important in career development.

In today’s complex and changing career development environment, individuals rely on a variety of social networks and information channels when seeking career advice and information. The social networks for counseling and the channels for obtaining career information show diverse characteristics, reflecting the multi-level and multi-dimensional needs of individuals in the process of career development. In terms of social networks for counseling, colleagues, friends and family members become the three most important objects of counseling, occupying 65.15, 67.20 and 51.68%, respectively. In particular, friends, with 459 choices, became the most frequently consulted, accounting for 67.20% of the total sample. In the process of career development, individuals are more inclined to trust informal social networks with which they have close relationships. In contrast, although superiors and industry experts provide valuable advice in some cases, the proportion of those who chose to consult them was only 27.23 and 27.82%, showing that formal sources of authoritative counseling do not dominate in the daily process of career counseling. The role of career mentors in career counseling should not be overlooked, with 48.32% choosing career mentors for counseling, showing their importance in guidance and career planning. Career mentors are able to provide more specialized and personalized career advice, which is especially important for those at a critical stage of career development. In terms of channels for obtaining career information, social media (e.g., LinkedIn, WeChat, etc.) and professional websites/forums have become the most popular channels, with 83.46 and 81.11% of people choosing these two channels, respectively. Social media, with its convenience and wide network coverage, makes information access easier and faster, while professional websites/forums satisfy professionals’ need for in-depth information through focused and specialized content. Although industry conferences are considered an important career information acquisition channel, only 32.21% chose this channel, which may be related to factors such as the frequency of organizing industry conferences, the cost of participation and the time schedule. Internal corporate announcements, on the other hand, had a 46.85% selection ratio, which indicates that employees are more likely to obtain career-related information in an environment with higher information flow and transparency within the organization. In the process of career development, individuals are more inclined to use social media and professional websites/forums, which are convenient, fast and have a wide coverage, to obtain information, and at the same time, rely more on close social networks such as friends and colleagues when consulting for career advice, which reflects the profound influence of the development of information technology and the popularity of social media on the process of career development, and reveals the actual behavioral patterns of individuals in career counseling and information acquisition.

## Conclusion

Individuals’ interests, career goals, and technological advances are the main sources of intrinsic motivation for career development. Individuals are enthusiastic and goal-driven in their career development, which keeps them motivated and engaged in the face of challenges. Self-efficacy frequently influences how people set their career goals, and having a higher level of self-efficacy can significantly boost a person’s confidence and perseverance in achieving their career goals. A company’s culture and development strategy have a significant impact on employees’ career development. By providing career training and internal promotion opportunities, companies can significantly increase employees’ career satisfaction and motivation. Supportive leadership and a people-centered corporate culture help stimulate employees’ intrinsic motivation and promote the achievement of career goals and career satisfaction. The rapid development of emerging industries, such as artificial intelligence, big data, new energy and other fields, provides abundant opportunities and motivation for career development, and individuals need to continuously learn and update their knowledge to remain competitive. Individuals in the process of career development acquire diverse information and resources through a wide range of social relationships, which contributes to the increase of career opportunities and career satisfaction. Individuals at the core of the career development process usually have extensive social networks and are able to quickly access important information about job vacancies, vocational training, and industry dynamics.

The gap between labor market demand and labor skill supply has led to difficulties in finding suitable talent for many jobs, while a large number of job seekers are unemployable due to inadequate skills. Labor market institutions and policies in some countries and regions may not be able to adapt in time to the rapid development of emerging industries, thus limiting career development, and overly strict labor market regulations a lack of flexibility may hinder the creation of new occupations and jobs. Social problems such as gender discrimination, age discrimination and racial discrimination can be obstacles to career development, and the influence of traditional cultural attitudes on career choices should not be ignored.

Through the provision of diversified vocational training programs and lifelong learning opportunities, the government and enterprises should work together to promote lifelong learning programs, provide flexible learning paths and diversified training courses to meet the learning needs of employees of different ages and occupational stages, and help employees upgrade their skills and adapt to the requirements of new technologies and new jobs. The government and enterprises need to work together to optimize the policy and institutional environment of the labor market, provide more flexible and inclusive employment policies, support the development of emerging occupations, create a fair and inclusive work environment, and gradually reduce or even eliminate gender, age and racial discrimination, so as to ensure that every employee has equal opportunities for career development. Developing online learning platforms, utilizing Internet technology to provide anytime, anywhere learning opportunities, helping employees upgrade their skills on their own, and supporting the development of various forms of employment such as part-time jobs, temporary jobs and freelancing, so as to inject more vitality into the labor market. Establishing a career change support system to provide career counseling, skills training and employment guidance to employees wishing to change careers, helping them make a smooth transition to a new career, and improving the social security system to ensure that all workers can enjoy basic social security in the process of changing careers, and to mitigate the risks and pressures of changing careers. Formulating and strictly enforcing anti-discrimination policies, ensuring fairness in recruitment, promotion and remuneration, and eliminating discrimination on the basis of gender, age and race. Conduct multicultural training to enhance employees’ understanding of and respect for different cultural backgrounds and promote teamwork and harmony within the organization. Provide employee support programs, including mental health counseling, career development guidance, etc., to help employees cope with work pressure and enhance professional well-being. Increase support for scientific and technological innovation, promote the research, development and application of new technologies, create more high-skilled jobs, and provide policy support and financial assistance for the digital transformation of enterprises to help them enhance their productivity and competitiveness in the process of transformation, while creating more digital jobs.

## Data Availability

The original contributions presented in the study are included in the article/supplementary material, further inquiries can be directed to the corresponding author.
